# Supplementary figures and images for: Warning: Humans cannot reliably detect speech deepfakes
Source: PLoS One. 2023 Aug 2;18(8):e0285333. doi: 10.1371/journal.pone.0285333 (PMC10395974; doi:10.1371/journal.pone.0285333)

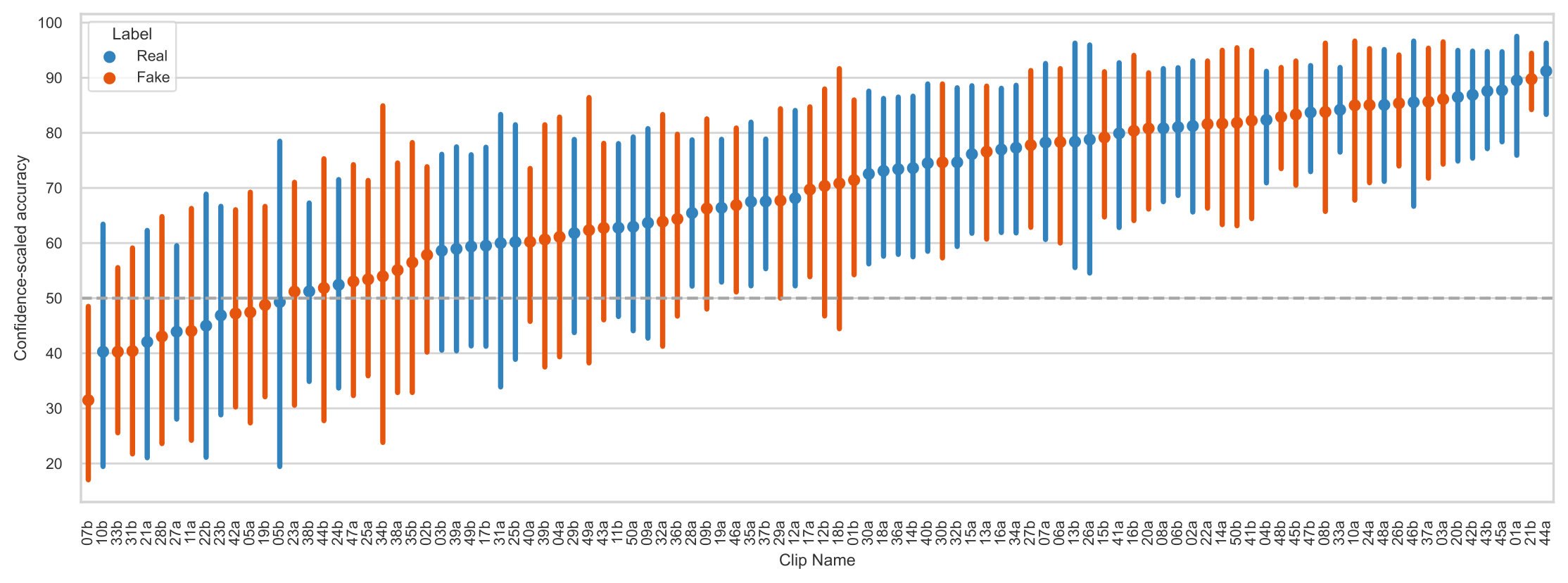

Supplement: S1 Fig — (TIF) [file pone.0285333.s001.tif]

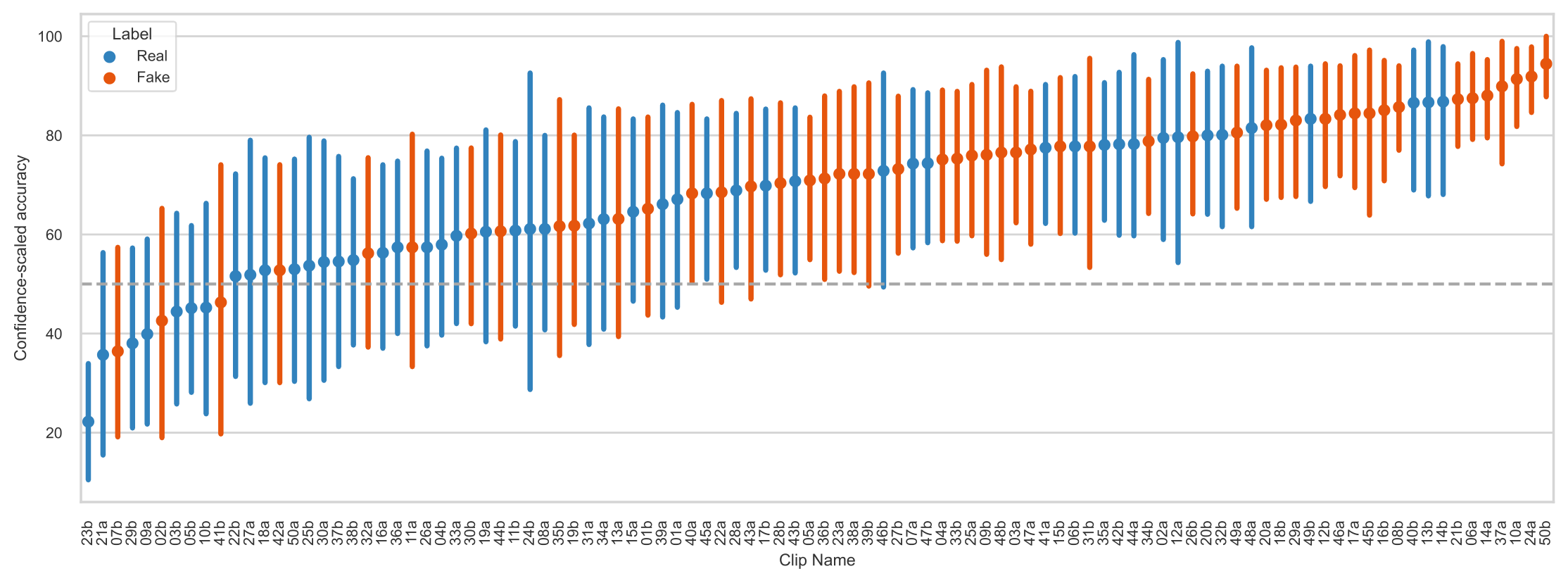

Supplement: S2 Fig — (TIF) [file pone.0285333.s002.tif]

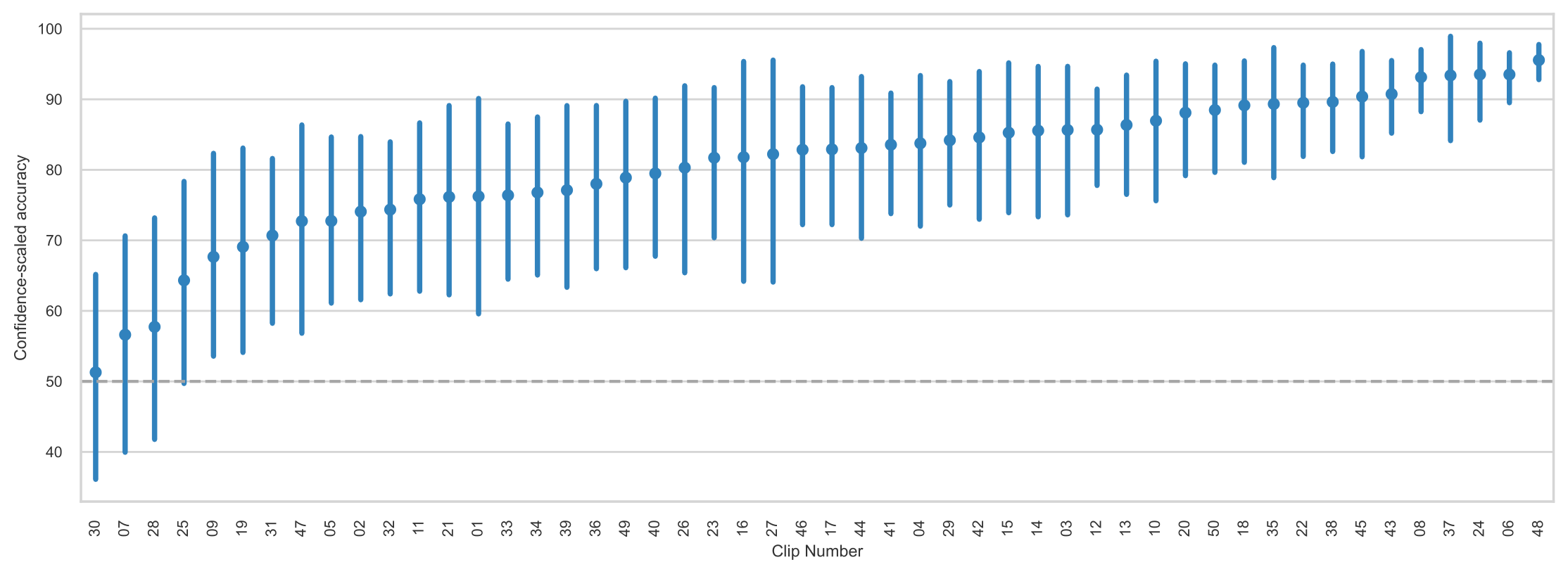

Supplement: S3 Fig — (TIF) [file pone.0285333.s003.tif]

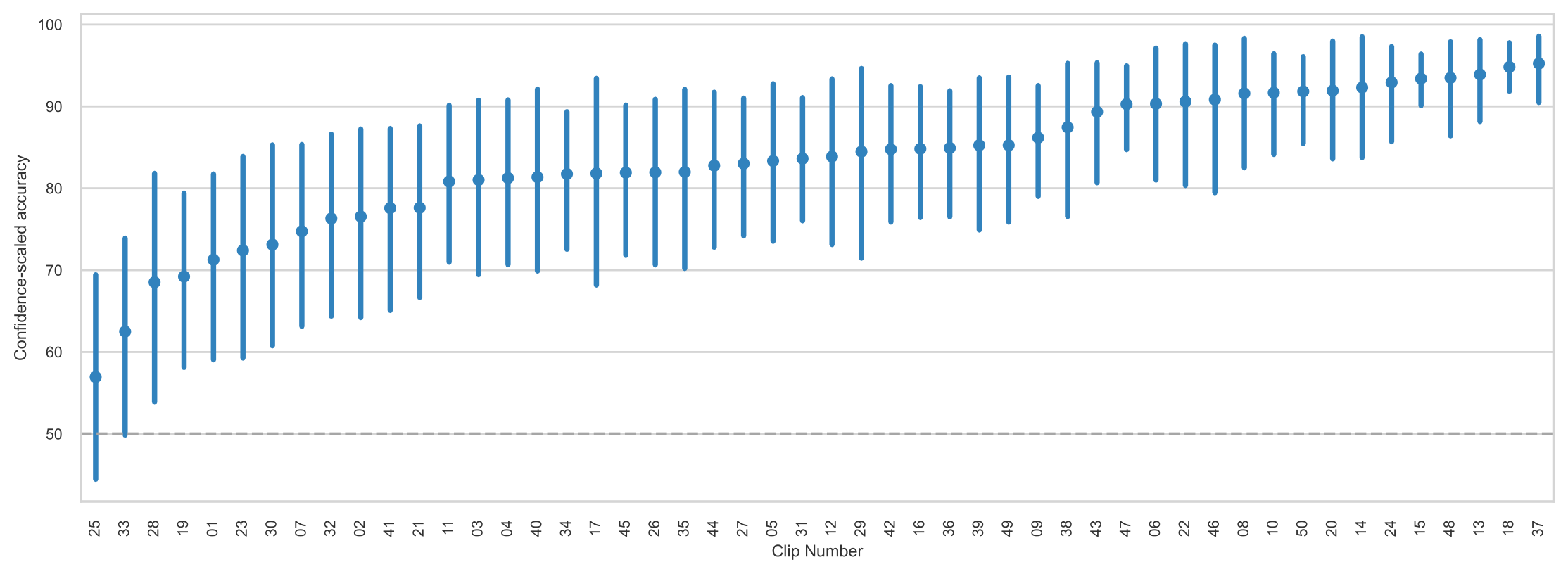

Supplement: S4 Fig — (TIF) [file pone.0285333.s004.tif]

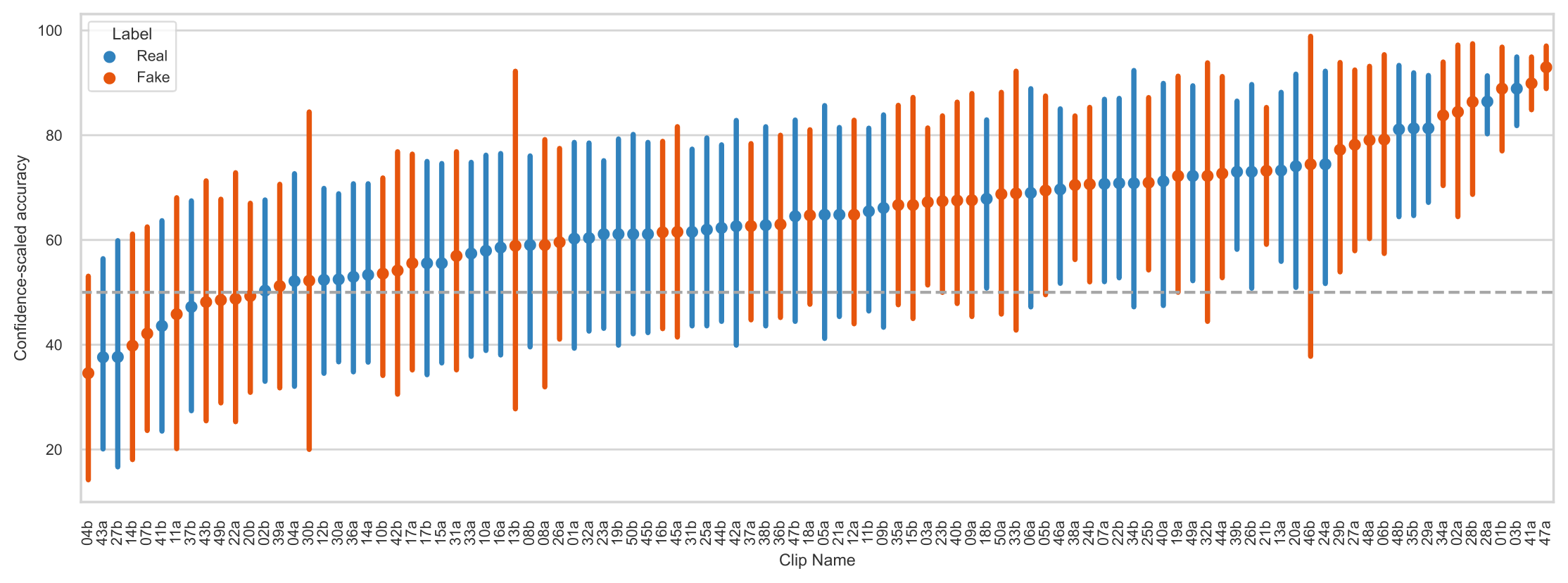

Supplement: S5 Fig — (TIF) [file pone.0285333.s005.tif]

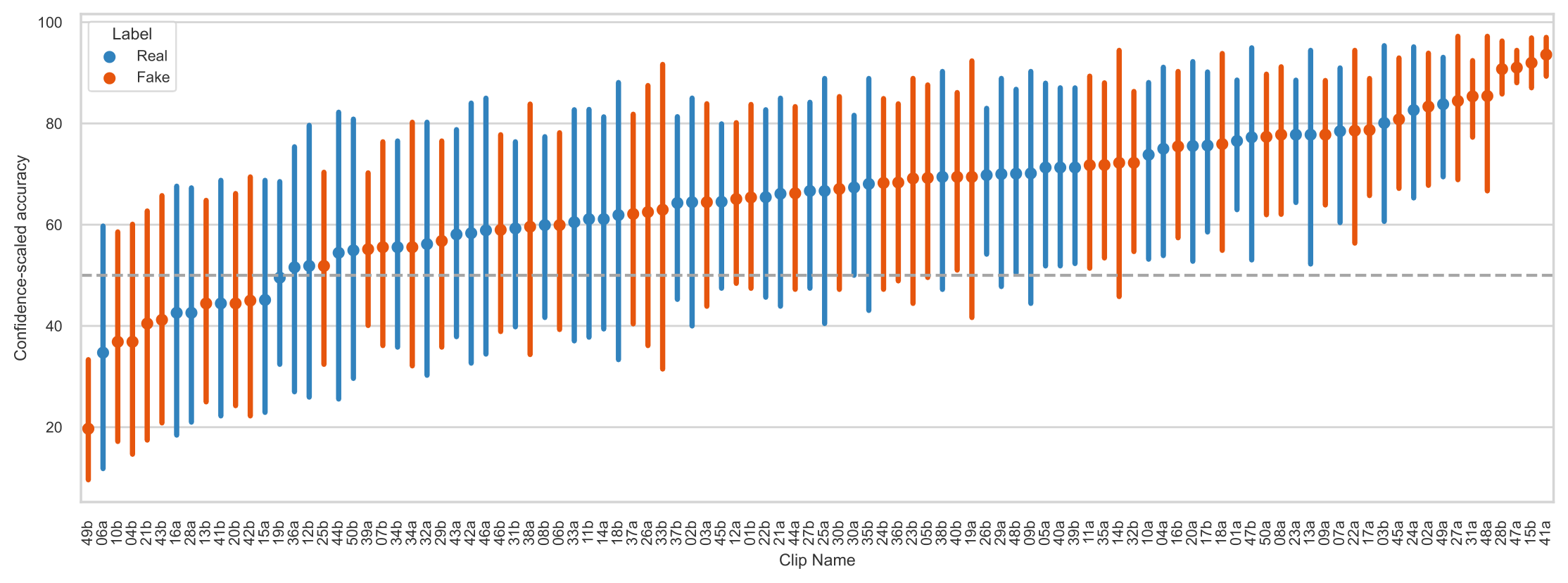

Supplement: S6 Fig — (TIF) [file pone.0285333.s006.tif]

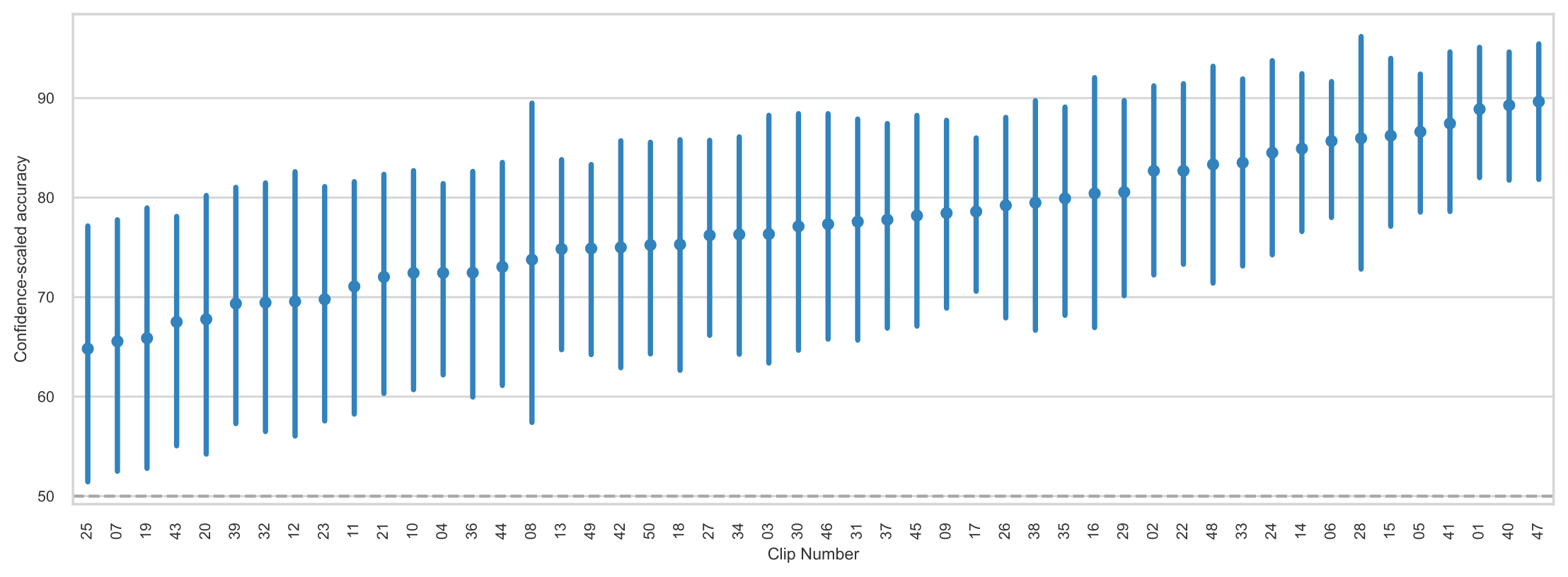

Supplement: S7 Fig — (TIF) [file pone.0285333.s007.tif]

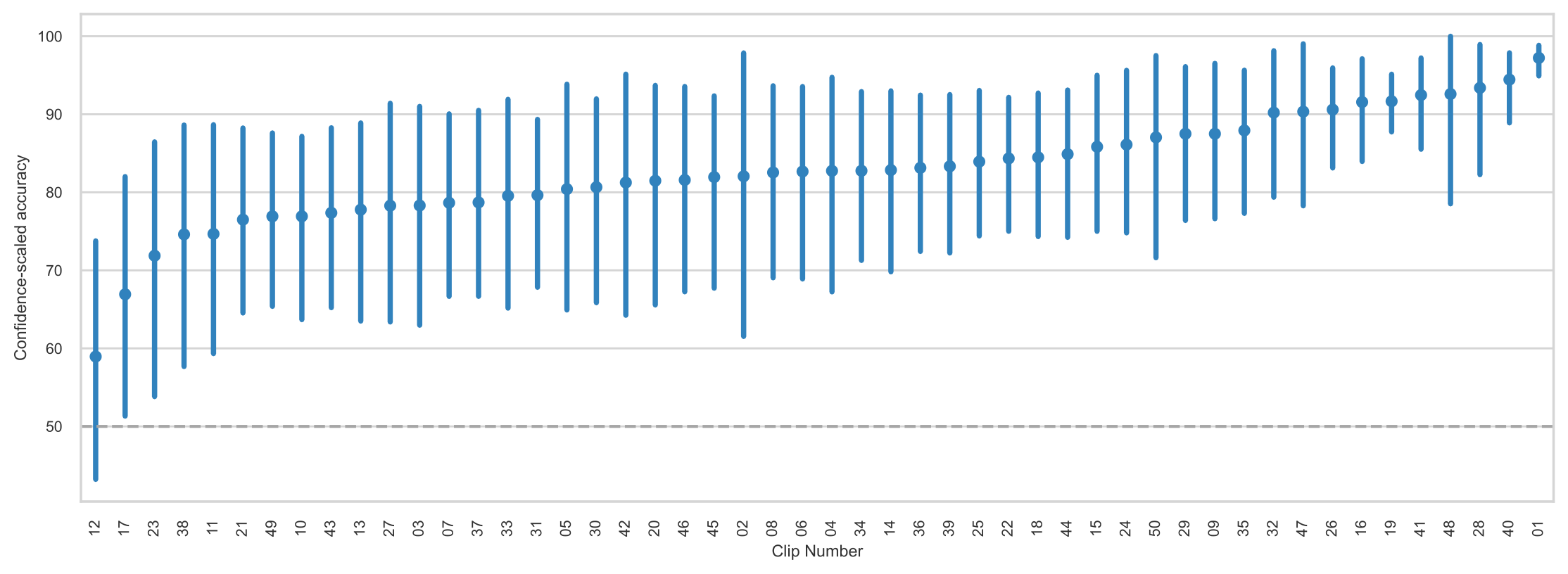

Supplement: S8 Fig — (TIF) [file pone.0285333.s008.tif]
